# Some Diseases of the Male Genital System

**Published:** 1908-02-22

**Authors:** 


### SOME DISEASES OF THE MALE GENITAL SYSTEM.

### IX.—TUBERCULOSIS OF THE TESTIS AND EPIDIDYMIS.

Last week we discussed the modes of infection of the testis by the tubercle bacillus and some points in the pathology of the disease. These points are not of academic interest only; they are important because they assist us in understanding its clinical course. Clinically tuberculous disease of the testis may be divided into two varieties, the acute and the chronic.

The acute form, sometimes known as "galloping phthisis of the testis," is the less common, and it will be discussed first. It generally starts with sudden pain in the scrotum. In many cases no apparent cause can be found, but others follow on an attack of gonorrhœal epididymitis; and, in fact, early acute tuberculosis of the testis resembles gonorrhœal epididymitis so closely that it is often difficult, or even impossible, to say definitely at what period in these cases one disease passes into the other. Very soon after the onset of pain the testis is found to be swollen and to have an irregular nodular outline, and if the case be watched from the beginning a fluctuating spot will be found in the course of a very few weeks, sometimes as few as two or three. The early formation of abscess is one of the most characteristic signs of acute tubercle of the testis. The accessory organs are attacked with astonishing rapidity, vas deferens, vesiculæ seminales, and even the prostate, and abscess formation may occur in any or all of these. Lastly, the condition is sometimes associated with a urethral discharge, which is not like a gonorrhœal discharge, but consists of thin sero-purulent material. It is explained as being probably due to tuberculous disease of the vesiculæ seminales or of the prostate. Tubercle bacilli can nearly always be found in it, if it is examined bacteriologically. As soon as the diagnosis of acute tuberculosis of the testis has been definitely made, the testis should be removed at the earliest possible opportunity with as much of the cord as is possible. No palliative measures hold out any hope of success. The very nature of the process is proof that the patient has no resistance to the tubercle bacillus, and delay will only result in the whole genital apparatus becoming progressively involved.

In the chronic form, which is also the usual one, the onset is insidious. An observant patient will possibly notice the presence of one or more nodular lumps in the epididymis before his attention is called to his condition by any definite symptom. At first these are quite painless, but later, when the whole epididymis and possibly parts of the testis are involved, pain of a dragging character is often complained of. This is probably partly due to the disease itself and partly to the increased weight of the organ. As in all conditions in which the testis is diseased, there may be, and probably is, some degree of hydrocele of the tunica vaginalis; it is rarely a large one, but simply a serous effusion as the result of the reaction of the tissues to the disease. It is exactly analogous to the pleural effusion, which often accompanies, or apparently precedes, pulmonary tuberculosis. It is now common knowledge that when an unexplained pleural effusion is found it is nearly always due to pulmonary tuberculosis, but the early diagnosis of this disease is so difficult a matter that the lesion, although present, does not give rise to sufficient physical signs to enable it to be detected. This is not the case in the testis, because by the time a hydrocele has made its appearance there is at any rate definite disease of the epididymis; but it should be remembered that the hydrocele, though only a slight one, may be sufficient to obscure the exact condition of affairs in the body of the testis. Sooner or later the hard nodules caseate and involve the skin over them. The length of time which occurs before this takes place is most variable. In the acute form we have seen that it may occur in a few weeks, but in the chronic as much as many months, or even a year may elapse. One or more fistulæ result, according to the number of abscesses which have been formed. Abscess formation is usually associated with increased pain, but this is relieved when the pus comes to the surface, just as it is in an acute inflammatory process. The after-history of an untreated case varies. In most cases the spontaneous opening which has been made by nature is insufficient to allow of the discharge of all the contents of the abscess, and, if the surgeon does not interfere, pus is continually discharged in small quantities, or the opening may heal over temporarily until the tension inside is again increased, when another eruption of pus takes place. In some cases, but unfortunately in very few, the initial evacuation of the abscess seems to enable the tissues to exert their natural bactericidal power, and the abscess cavity is cured by connective tissue reaction and subsequent fibrosis.

Finally, as in the acute variety, the more distant parts of the genital apparatus may be involved, and these cases should always be examined by the rectum to see if the prostate is diseased. If it is, it will be felt to be enlarged, distinctly differentiated from the surrounding structures, and to present to the examining finger either hard nodules or a soft fluctuating spot. In the former case no surgical procedures are practicable, but if there be a prostatic abscess it should be opened by means of a transverse perineal incision, through which the urethra is separated from the rectum, until the abscess cavity is reached and the pus can be evacuated.

In some cases both testes are involved in the disease, and when this occurs it generally happens that the second one is attacked a considerable time after the other, in some cases so long after that the disease of the second testis must be regarded as a new and distinct outbreak of tuberculosis rather than as a local extension of the original disease. The writer has seen two cases in which the remaining testicle became tuberculous several years after the first one, and in spite of the fact that castration was performed at an early stage; in fact, in one case it was removed immediately because it was supposed to be affected with malignant disease.

